# Identifying Core Competencies for Prefieldwork Occupational Therapy Students in Adult Therapy: A Delphi Study With Clinical Therapists

**DOI:** 10.1155/oti/2127249

**Published:** 2025-12-09

**Authors:** Shih-Chen Fan, Jia-Yu Shao, Ting-Yu Lu, Wen-Chou Chi, Chia-Hui Hung

**Affiliations:** ^1^ Department of Occupational Therapy, College of Medical Science and Technology, Chung Shan Medical University, Taichung City, Taiwan, csmu.edu.tw; ^2^ Occupational Therapy Room, Chung Shan Medical University Hospital, Taichung City, Taiwan, csh.org.tw; ^3^ Department of Public Health, Chung Shan Medical University, Taichung City, Taiwan, csmu.edu.tw

**Keywords:** curriculum development, Delphi method, occupational therapy education, prefieldwork competency, professional readiness

## Abstract

**Introduction:**

Prefieldwork students often experience challenges transitioning from academic learning to clinical practice due to gaps in competency development. While established frameworks define postgraduate competencies, the specific needs of prefieldwork students remain underexplored. This study is aimed at identifying core competencies essential for prefieldwork students in adult therapy settings.

**Method:**

A two‐phase study design was employed. In Phase 1, focus group discussions involving clinical supervisors, faculty members, and prefieldwork students identified key competency domains. In Phase 2, a modified Delphi method was conducted with clinical supervisors (*n* = 22 in Round 1, *n* = 20 in Round 2) to establish consensus on competency inclusion and definitions. Data analysis included thematic coding, interrater reliability assessment (Cohen′s kappa), and statistical evaluation using mean scores, interquartile range (IQR), and content validity ratio (CVR).

**Results:**

The focus group discussions identified key competency challenges, including insufficient knowledge of splinting principles, limited assessment skills, and difficulties in professional communication. Initial thematic analysis mapped these challenges to nine established competency domains. The Delphi survey further added five emerging competencies: *reflection*, *coping strategies*, *resource linking*, *multiple perspectives*, and *local–global awareness*. Five emerging competencies were organized into three thematic groups: interpersonal adaptation, contextual cognition, and system navigation. However, the management competency was excluded due to low consensus.

**Conclusion:**

This study highlights critical competency gaps in prefieldwork OT education, emphasizing the need for structured curriculum interventions. Prefieldwork OT curricula should strengthen coping, reflection, and resource linking through reflective journaling, stress management, and resource planning. The role of local–global awareness should be clarified. The management competency was excluded from the final competency list due to limited appropriateness.

**Key Findings**

• Prefieldwork students need improved coping, reflection, and resource‐linking skills, which can be developed through reflective journaling, stress management modules, and proactive resource planning.
• *Local–global awareness* had mixed consensus, suggesting the need for clearer definition in prefieldwork education.
• *Management* was excluded due to low agreement on its relevance for prefieldwork students.

## 1. Introduction

Competency‐based medical education (CBME) has become a dominant approach in health professional education, emphasizing the attainment of essential core competencies required for clinical practice [1]. In occupational therapy education, bridging the gap between academic training and clinical practice is crucial to ensure that students develop the necessary professional skills before entering real‐world practice. In countries such as Australia, New Zealand, Taiwan, and the United Kingdom, the bachelor′s program serves as the primary entry pathway for occupational therapists [2, 3]. Although these programs have established core competency frameworks aligned with the minimum standards set by the World Federation of Occupational Therapists (WFOT), they predominantly emphasize competencies expected upon completion of fieldwork and graduation. Understanding this gap is essential for refining occupational therapy curricula and ensuring that students receive adequate preparation. However, the specific competency needs of prefieldwork students remain underexplored.

Within occupational therapy education, students progress through distinct learning phases, typically categorized into prefieldwork academic education and clinical fieldwork. For example, in Taiwan, prefieldwork education in the first 3 years focuses on theory and foundational skills. In contrast, the full‐time internship in the 4th year presents a stark difference in both content and context compared to the preceding years of study [4]. Clinical fieldwork demands real‐time decision‐making, interdisciplinary collaboration, and direct patient care [5]. These disparities may result in inconsistent levels of preparedness among students entering fieldwork.

Internationally, medical and health education programs have implemented prefieldwork training models to address similar gaps. For example, medical schools in Australia offer structured procedural skills courses with individualized feedback to enhance clinical readiness [6]. Similarly, the clinical apprenticeship model in New Zealand integrates academic learning with supervised clinical exposure, bridging the transition from medical student to intern [7]. While these initiatives have demonstrated effectiveness in medical education, prefieldwork competency development remains underexamined in occupational therapy programs, with limited frameworks available to assess students′ readiness for fieldwork.

While prefieldwork training models have been explored in other health professions, the specific competencies required of occupational therapy students before fieldwork remain unclear. To address this gap, this study identifies core competencies expected in adult rehabilitation settings, aiming to inform curriculum design and enhance students′ clinical preparedness.

## 2. Materials and Methods

**Figure 1:**
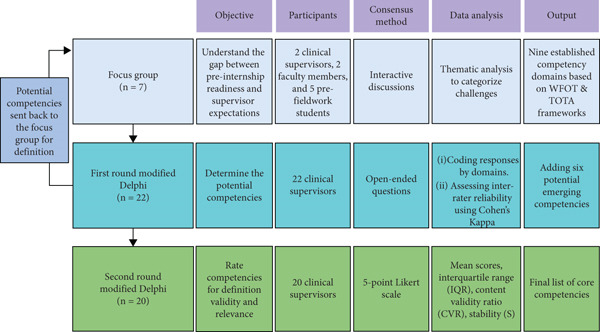
CONSORT study design flowchart with participant numbers.

This study was carried out in two phases: a focus group and a Delphi survey. Figure 1 summarized the study process. The research protocol was reviewed and approved by the Institutional Review Board of Chung Shan Medical University, under Approval Number CS2‐24151. All participants provided written informed consent.

### 2.1. Focus Group Discussions

#### 2.1.1. Purpose and Participant Selection

This study employed focus group discussions to identify challenges encountered by third‐year occupational therapy students transitioning to clinical practice and to explore the expectations of clinical supervisors. Focus groups were chosen as they allow for interactive discussions, generating insights that may not emerge in individual interviews [8]. Participants were recruited using convenience sampling with eligibility criteria. Eligible participants were as follows: third‐year OT students who had completed coursework in adult therapy. Faculty members were eligible if they taught occupational therapy courses. Clinical supervisors were eligible if they were currently supervising students in adult therapy. Recruitment of students was through a campus bulletin board announcement. Participation was voluntary. A complimentary lunch was provided. To minimize potential power imbalances between students, faculty, and clinical supervisors, students were informed that the focus group was only for course improvement. Their responses would not affect course grades.

A single heterogeneous focus group was conducted. The group included five prefieldwork students, two faculty members, and two clinical supervisors.

#### 2.1.2. Data Collection and Analysis

**Figure 2:**
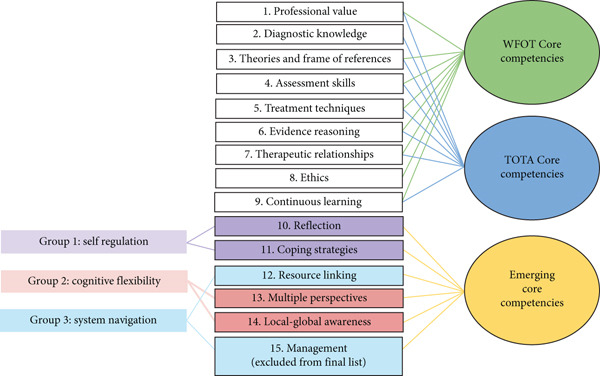
Fifteen potential competencies noted after Delphi Round 1.

A trained member of the research team moderated the discussions. The moderator had completed a workshop focused on questioning skills, maintaining discussion flow, and fostering a warm, neutral, nonjudgmental environment. The moderator encouraged participants to express their views. The discussion lasted 120 min, was audio‐recorded, and transcribed verbatim for analysis. Transcripts would not include names or any identifiers. A thematic analysis was then conducted to categorize identified challenges into competency domains. These domains were mapped to existing competency frameworks established by the WFOT and the Taiwan Occupational Therapy Association to ensure alignment with both international and local professional standards. The resulting domains and their definitions served as the foundation for developing the Delphi survey. The nine domains were as follows: *professional value*, *diagnostic knowledge*, *theories and frame of references*, *assessment skills*, *treatment techniques*, *evidence reasoning*, *therapeutic relationships*, *ethics*, and *continuous learning* (Figure 2).

### 2.2. Overview of the Delphi Approach

The study followed a modified Delphi approach consisting of two rounds, which is consistent with prior research indicating that two to three rounds are generally sufficient to achieve expert consensus in healthcare‐related studies (Hsu & Sandford, 2007).

### 2.3. Round 1: Open‐Ended Survey

In Round 1, clinical supervisors completed an open‐ended questionnaire to elicit essential competencies expected of students across nine domains. A minimum of three competencies was requested for each domain. Open‐ended surveys could ensure comprehensive expert input before moving to structured rating scales [9].

#### 2.3.1. Participant Selection

A purposive sampling was used to recruit clinical supervisors from 24 affiliated fieldwork sites. Eligible participants were occupational therapy supervisors specializing in adult therapy, with at least 3 years of Level II fieldwork supervision experience. Surveys were distributed via email and printed mail. The survey remained open for 1 month, with a reminder sent mid‐period.

#### 2.3.2. Data Analysis

Responses were segmented into discrete competency statements by two independent coders and categorized according to pre‐established domains. To ensure consistency in coding, a preliminary 1.5‐h consensus meeting was conducted before formal analysis.

**Figure 3:**
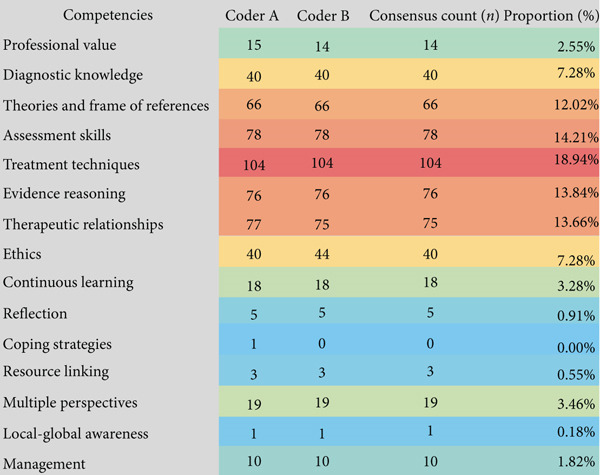
Correspondence between coders and core competencies in Delphi Round 1. *Note:* Proportion (%) = consensus count (*n*) of one competency/total counts. Red indicates higher agreement; blue indicates lower agreement.

Interrater reliability was assessed using Cohen′s kappa coefficient (*κ*) [10, 11]. Kappa scores were interpreted as follows: *κ* ≤ 0, no agreement; 0.01–0.20, slight agreement; 0.21–0.40, fair agreement; 0.41–0.60, moderate agreement; 0.61–0.80, substantial agreement; 0.81–1.00, almost perfect agreement. A threshold of *κ* > 0.61 was set to indicate sufficient agreement for competency classification [6]. After independent coding, the Cohen′s kappa coefficient between two coders was calculated as *κ* = 0.716, indicating substantial interrater reliability [10] (Figure 3). The validated competency list was then used to develop the second‐round Delphi questionnaire.

### 2.4. Round 2: Structured Rating Survey

#### 2.4.1. Survey Content

In Round 2, clinical supervisors were asked to evaluate each competency based on their satisfaction with competency definitions and the appropriateness of their inclusion in the final framework.

Each definition was rated on a 5‐point Likert scale, from 1 (*strongly disagree*) to 5 (*strongly agree*). For example, participants were presented with the definition of professional value as “recognizing the principles of occupational therapy and delivering client‐centered care within available resources.” They were then asked to rate its clarity and validity. Supervisors rated the appropriateness using a 5‐point Likert scale, with reference to the frequency with which each competency was identified in Round 1.

#### 2.4.2. Participant Selection

The same group of clinical supervisors who participated in Round 1 was reinvited.

#### 2.4.3. Data Analysis

In Round 2, each competency was assessed using the following metrics: mean (M) score, indicating the central tendency of supervisor ratings; standard deviation (SD), measuring rating variability; interquartile range (IQR), quantifying consensus; and IQR ≤ 1, indicating high agreement [12].

Content validity ratio (CVR) evaluates the proportion of experts who rated a competency as essential, using the following formula: (*n* *e* − *N*/2)/(*N*/2) where *n* *e* represents supervisors′ rating the competency as “important” (Likert 4–5) and *N* is the total number of respondents [13]. Stability (*S*) measures response consistency across rounds, which is defined as SD/M. *S* ≤ 0.8 indicated high consensus, negating the need for further Delphi rounds [14, 15]. The final competency retention was based on a three‐factor decision rule: M score ≥ 4.0, indicating strong agreement; IQR ≤ 1, suggesting low variability in expert ratings; and CVR ≥ 1, confirming consensus on importance [11, 15]. Competencies failing to meet all three criteria were excluded from the final framework.

## 3. Results

### 3.1. Demographic Characteristics

The focus group discussion included two clinical supervisors, two university faculty members, and five prefieldwork students. One clinical supervisor had 24 years of clinical experience, specializing in hand trauma rehabilitation, work hardening, and splint fabrication. The other had 25 years of experience and was the head of adult therapy at a medical center. The two university faculty members were associate professors with teaching expertise in long‐term care and adult occupational therapy. They had an average of 17.5 years of teaching experience (SD = 3.54). The five prefieldwork students had an average age of 21.8 years (SD = 0.84).

### 3.2. Challenges Mapped to Competency Domains

Several common challenges faced by prefieldwork students emerged from the discussions. Many participants noted that students lacked sufficient knowledge of splinting and struggled to apply the techniques flexibly. Additionally, gaps were identified in students′ familiarity with evaluation procedures. Language barriers, particularly limited proficiency in Taiwanese dialects, were also highlighted. Moreover, coping strategies were a common concern, as students often felt stressed from low confidence during fieldwork.

The identified challenges were categorized into the nine domains in the Methods section. The detailed definitions of these competency domains are presented in Appendix 1.

### 3.3. Survey Response Rates and Participant Demographics

The response rates and professional backgrounds of clinical supervisors participating in the Delphi survey are summarized in Table 1. In the first round, 24 questionnaires were distributed, with 22 valid responses received (response rate: 91.67%). In the second round, 20 valid responses were collected (response rate: 83.33%).

**Table 1:** Response rates and backgrounds of clinical supervisors in the Delphi survey.

**Variables**		**Round 1** **n** **(%)**	**Round 2** **n** **(%)**
Total response rates		22 (91.67%)	20 (83.33%)

Types of settings	Medical center	11 (50%)	10 (50%)
	Regional hospitals	6 (27.27%)	5 (25%)
	District hospitals	2 (9.09%)	2 (10%)
	Community based/others	3 (13.64%)	3 (15%)

Number of clinical supervisors (*n*)	≧ 16 persons	2 (9.09%)	0 (0%)
	11–15 persons	2 (9.09%)	2 (10%)
	6–10 persons	9 (40.91%)	9 (45%)
	< 5 persons	9 (40.91%)	9 (45%)

Number of Fieldwork Level II students supervised annually	≧ 16 persons	1 (4.55%)	1 (5%)
	11–15 persons	0 (0%)	0 (0%)
	6–10 persons	4 (18.18%)	2 (10%)
	< 5 persons	17 (77.27%)	17 (85%)

Among the clinical supervisors in the first round, 50% were from medical centers and 27.3% from regional hospitals. Regarding the number of clinical supervisors per setting, 40.91% reported having between 6 and 10 supervisors, while another 40.91% reported fewer than five. When asked about the number of Fieldwork Level II students they supervised annually, 77.27% indicated that they supervised fewer than five students.

### 3.4. Evaluation of Competency Definitions and Appropriateness

Based on the feedback from the Delphi survey, expert members of the focus group noted that certain competencies extended beyond the previously defined nine domains. Consequently, this study further categorized six emerging competency domains: *reflection*, *coping strategies*, *resource linking*, *multiple perspectives*, *local–global awareness*, and *management*. These six competencies, together with the previously defined nine, constitute a total of 15 potential competencies (Figure 2).

### 3.5. Final Competency Framework Refinement

**Figure 4:**
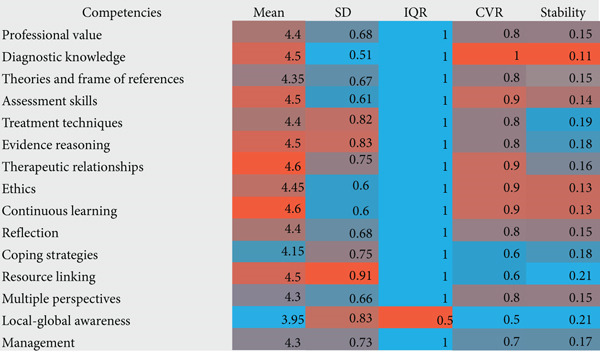
Satisfaction scores in the second round of Delphi Round 2. *Note:* Red indicates higher satisfaction; blue indicates lower satisfaction.

The second round of the modified Delphi survey assessed clinical supervisors′ satisfaction with competency definitions and the appropriateness of their inclusion in the final framework. The satisfaction scores for each definition are detailed in Figure 4. The highest satisfaction consensus, in order, are *therapeutic relationships* (M = 4.6, CVR = 0.9), *continuous learning* (M = 4.6, CVR = 0.9), *diagnostic knowledge* (M = 4.5, CVR = 1), and *assessment skills* (M = 4.5, CVR = 0.9).

A union‐based deletion criterion was applied, requiring a competency to meet all three exclusion conditions in order to be excluded. As a result, no competencies were eliminated solely based on definition satisfaction. However, *local–global awareness* received relatively lower M scores and CVR values, while *coping strategies* and *resource linking* also exhibited lower CVR values. This suggests that, although these competencies were not removed, clinical supervisors expressed relatively lower satisfaction with their definitions.

**Figure 5:**
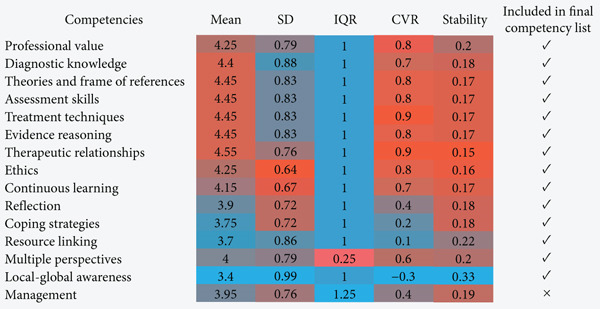
Appropriateness score in the second round of Delphi Round 2. *Note:* Red indicates higher appropriateness; blue indicates lower appropriateness.

The appropriateness scores indicate that *therapeutic relationships* (M = 4.55, CVR = 0.9), *assessment skills* (M = 4.45, CVR = 0.8), *treatment techniques* (M = 4.45, CVR = 0.9), and *theories and frame of references* (M = 4.45, CVR = 0.8) received the highest appropriateness ratings (Figure 5). However, *management* meeting the exclusion threshold received a M score of 3.95, an IQR of 1.25, and a CVR of 0.4. Consequently, this competency was excluded from the final list, leaving 14 core competencies.

## 4. Discussion

This study used focus group discussions and a modified Delphi method to establish consensus on the core competencies of prefieldwork occupational therapy students in adult therapy. Through expert validation and systematic analysis, 14 competencies were finalized. The next section will focus on the two competencies that achieved the highest appropriateness consensus: *therapeutic relationships* and *assessment skills*. To enhance conceptual coherence and reduce redundancy, five emerging competencies are organized into three thematic groups: interpersonal adaptation, contextual cognition, and system navigation. One excluded competency, *management*, is also addressed.

Strong *therapeutic relationships* foster clients′ confidence and motivation [16]. Quotes from clinical supervisors emphasized that “students who demonstrate active listening, build trust, and use client‐friendly language” can enhance client participation. Clients who feel supported and respected demonstrate greater commitment to rehabilitation. Therefore, integrating specific training on therapeutic relationships into the future curriculum is necessary. The Intentional Relationship Model (IRM) provides a structured approach for therapists to adapt interpersonal strategies based on client needs [17], while role‐playing and reflective learning further strengthen these competencies [18].

*Assessment skills* involve selecting and administering standardized or nonstandardized assessments. However, the literature suggests that focusing solely on the reliability and validity of measurement tools or confining evaluation to fragmentary “physical component measurements” often fails to encompass the holistic occupational performance within diverse cultural and environmental contexts [19, 20]. As such, strengthening assessment skills to integrate both “objective measurement” and “meaning‐driven” approaches has become a critical issue for occupational therapy education [21]. Quotes from clinical supervisors also highlighted “interpreting assessment results tailored to the patient′s individual needs” as highly important. The curriculum may incorporate training in interpreting assessment outcomes in diverse, real‐life contexts [19, 20]. Context‐based teaching strategies, such as clinical reasoning and simulation, could be helpful [22].

### 4.1. Group 1: Interpersonal Adaptation

The first group of emerging competencies was interpersonal adaptation, which includes *reflection* and *coping strategies.* Both reflection and coping strategies involve self‐awareness and adaptive responses to interpersonal challenges. Based on feedback from clinical supervisors, *reflection* encompasses “reflecting on treatment effectiveness,” “recognizing one′s professional capabilities,” and “using reflection frameworks to improve systematic decision‐making.” Therapists who reflect on their own behaviors, thoughts, and emotions are better positioned to make improvements in therapeutic strategies. Reflection also fosters continual self‐directed learning [23, 24].

*Coping strategie*s, on the other hand, are defined as “managing stress and sustain emotional resilience through support‐seeking and problem‐solving.” One quote from clinical supervisors is “students who can proactively seek help demonstrate stronger coping capacity.” This perspective is consistent with past research findings. Research indicates that problem‐focused and emotion‐focused strategies are commonly employed by students, although some still engage in avoidant methods such as self‐blame [25, 26].

While reflection enhances insight, coping strategies enable emotional resilience. Together, they equip students with the interpersonal adaptation. The future curriculum could incorporate structured reflective journaling, self‐assessment tools, proactive resource planning, and stress management modules to strengthen *reflective* and *coping strategies* [27].

### 4.2. Group 2: Contextual Cognition

The second group of emerging competencies was contextual cognition, which encompasses *multiple perspectives* and *local–global awareness*. Together, these competencies enable students to navigate both individual, social, and systemic contexts. One quote from clinical supervisors is to “respect individuals regardless of their culture, beliefs, age, race, sexual orientation, functional abilities, or socio‐economic status.” Lesbian, gay, bisexual, and transgender (LGBT) individuals often conceal their gender identity or sexual orientation because of inadequate acceptance by family or peers [28]. Moreover, culturally insensitive healthcare systems discourage LGBT individuals from seeking medical resources [29]. Therefore, the future curriculum should enhance students′ understanding of LGBT populations and sexual orientation equity, adopting a respectful and open attitude to promote occupational justice.

Understanding diverse perspectives also extends to family roles across the life course. In the post–pandemic era, the proportion of adult children living with parents has risen; thus, the needs of family members remain an important consideration for therapists [30]. These examples show that contextual cognition involves understanding both personal identities, like sexual orientation, and wider family and social dynamics.

In addition, contextual cognition also involves *local–global awareness*. This includes considering how local and global systems shape occupational participation. One quote from clinical supervisors is “students should possess knowledge of both local and international long‐term care systems.” This competency could be demonstrated when a student identifies how Taiwan′s aging‐in‐place policy influences older adults′ access to home‐based services and compares it with the policy in other countries. However, this competency received mixed ratings from clinical supervisors. While some supervisors emphasized the importance of understanding the broader occupational therapy landscape, others viewed international exposure as less immediately relevant to students entering local clinical practice. This finding is consistent with previous research indicating that knowledge of global healthcare trends enhances professional adaptability but may not be a priority at the prefieldwork stage [31]. Given the increasing globalization of healthcare, it may be beneficial to further explore how this competency can be effectively integrated into prefieldwork curricula.

By integrating awareness of individual identities, family dynamics, and broader local–global environments, contextual cognition enables students to deliver context‐sensitive services.

### 4.3. Group 3: System Navigation

The third group of emerging competencies is system navigation, which includes *resource linking* and, to a lesser extent, *management skills*. This competency involves helping clients access appropriate healthcare, financial, and community resources to support their occupational participation.

*Resource linking* involves navigating financial and logistical resources and coordinating referrals to optimize patient outcomes. One quote from clinical supervisors is “to utilize professional resources to provide occupational therapy services that serve the client′s best interests.” Effective resource allocation enhances service efficiency and improves cost‐effectiveness. For example, in occupational therapy, clients may require various resources, including assistive device subsidies, home therapy grants, home modification funding, and long‐term care transportation services. These resources span different government agencies. In the case of Taiwan, assistive device subsidies are divided into long‐term care subsidies and disability subsidies, managed by the Health Bureau and Social Affairs Bureau, respectively. The eligibility criteria, application process, and subsidy amounts vary, making it essential for fieldwork students to be familiar with these resources. Strategies for integrating resources include understanding eligibility and staying in contact with providers. Therapists should assess client needs and clarify what each resource offers. Building strong ties with key providers and setting clear agreements can improve coordination. We believe that occupational therapy interns must be well‐versed in resource‐linking strategies to provide comprehensive services. Therefore, resource linking remains a crucial competency that should not be overlooked in their curriculum.

Although *management* was initially considered during competency development, it was ultimately excluded from the final list due to limited consensus. This implies that clinical settings prioritize students′ professional skills and practical performance over management abilities. Alternatively, supervisors may equate “management skills” with “leadership skills,” leading to less emphasis on management competencies. Literature on management skills in clinical education is relatively limited, and even when time management is covered, it receives minimal discussion [32, 33].

Current research primarily focuses on students′ professional skills, communication, and problem‐solving abilities during clinical fieldwork. However, management competency was not entirely irrelevant for fieldwork students. Although students in teaching hospitals may have limited exposure to higher level management tasks such as workforce allocation, resource distribution, and financial management, they still encounter basic management responsibilities, such as organizing materials and maintaining the clinical environment [34]. In community‐based fieldwork, students are more likely to engage in management‐related tasks due to staffing constraints [35]. Therefore, students should acquire basic management skills before their fieldwork to ensure a smoother transition. Future research could further explore whether excluding management skills from clinical training is justified.

## 5. Conclusion

In conclusion, the inclusion of emerging competencies alongside traditional domains reflects the expanding scope for curriculum development in occupational therapy education. As healthcare systems continue to evolve, there is a growing need for occupational therapists who possess not only technical expertise but also the ability to navigate cultural, interpersonal, and systemic factors that influence patient care. Future curriculum enhancements could focus on three key areas: First, integrating *reflection* and *coping strategies* into academic coursework to better prepare students for clinical challenges; Second, incorporating personal, social, local, and global healthcare perspectives in professional training to enhance adaptability and awareness of diverse practice settings; and Third, expanding opportunities for resource linking and interdisciplinary collaboration to help students develop practical skills in navigating healthcare systems.

## Disclosure

No persons or third‐party services were involved in the research or manuscript preparation other than those listed as authors or acknowledged.

## Conflicts of Interest

The authors declare no conflicts of interest.

## Funding

This study was funded by Chung Shan Medical University within the framework of the Higher Education Sprout Project by the Ministry of Education (1‐6‐10, 2024).

## Data Availability

The data that support the findings of this study are available on request from the corresponding author. The data are not publicly available due to privacy or ethical restrictions.

## Appendix 1

**Table A1:** Definition of core competencies.

**Core competency**	**Definition**	**References**
1. Professional value	A deep understanding of occupational therapy′s distinct and essential role within the healthcare system, a commitment to helping clients through client‐centered practice, and the effective utilization of available resources to deliver services that maximize client well‐being are fundamental to its professional values	[36–38]
2. Diagnostic knowledge	A comprehensive understanding of commonly encountered conditions, including their diagnosis, etiology, symptoms, prognosis, overall disease course, treatment processes, and recovery outcomes	[39–41]
3. Theories and frame of references	Familiar with discipline‐specific theories and technical knowledge, including anatomy, physiology, medical imaging, and key models and frameworks in physiological, psychological, and pediatric domains, understanding their principles, applications, and target populations	[42, 43]
4. Assessment skills	The ability to effectively use assessment tools and methods encompasses selecting appropriate tools based on clinical rationale, administering standardized and nonstandardized assessments, and adapting assessment approaches according to client abilities and practice settings	[43, 44]
5. Treatment techniques	Knowledge and application of therapeutic techniques, including activity design and analysis, formulation of treatment goals, splint fabrication, environmental modifications, and provision of transition services	[45]
6. Evidence reasoning	In clinical decision‐making, the ability to incorporate clinical experience, research evidence, and client values into professional reasoning. This includes procedural, interactive, and pragmatic reasoning, encompassing all cognitive processes involved in the therapeutic process, such as logical thinking, problem solving, and critical thinking	[2, 46]
7. Therapeutic relationships	The ability to integrate skills, attitudes, and approaches to establish a therapeutic relationship with clients, while engaging in interprofessional communication based on client needs and professional expertise. This includes respecting and collaborating with team members to adapt leadership concepts within the team	[47, 48]
8. Ethics	Possessing personal values and attitudes—such as empathy, respect, dignity, compassion, honesty, creativity, and individual characteristics—enables the application of appropriate ethical principles in therapy. These principles include altruism, nonmaleficence, autonomy and confidentiality, social justice, procedural justice, integrity, and fidelity	[49]
9. Continuous learning	Engaging in purposeful and lifelong learning of skills and knowledge while maintaining innovation	[50, 51]
10. Reflection	The ability to develop a deep understanding of one′s own state, including emotions, strengths, weaknesses, thoughts, behaviors, needs, and motivations. This competency involves engaging in critical self‐reflection on personal performance within broader contexts to facilitate continuous learning and professional growth	[52, 53]
11. Coping strategies	Managing stress and sustain emotional resilience through support seeking and problem solving	[54, 55]
12 Resource linking	The ability to identify, apply, and network available internal and external resources to meet client needs. This includes coordinating transition services like discharge planning and special education, as well as navigating healthcare, financial, and logistical resources to optimize patient outcomes	[56, 57]
13. Multiple perspectives	The ability to understand diverse cultural backgrounds and societal viewpoints, respect clients′ preferences or cultures, and demonstrate concern for clients in a “whole person” manner	[58, 59]
14. Local and global awareness	For understanding occupational therapy in domestic or international contexts, such as employment directions, workplace environments, and professional opportunities in the field of occupational therapy	[60]
15. Management	Management involves planning, organizing resources, leading, controlling, and coordinating various aspects, including time management, risk management, administration, and operational management within the field of occupational therapy	[50]
